# A Facile Nanocarrier for Paclitaxel Delivery Based on Carboxymethyl Chitosan Encapsulated 6-Deoxy-6-Mercapto-β-Cyclodextrin Grafted Concave Cubic Gold

**DOI:** 10.3390/nano16060378

**Published:** 2026-03-21

**Authors:** Hao Li, Lin Zhang, You Long, Chao Shen, Song Zhang, Fang Chen, Nan Chen, Chenghong Huang

**Affiliations:** 1 School of Chemistry and Chemical Engineering, Chongiqng University of Science and Technology, Chongqing 401331, China; 2023205009@cqust.edu.cn (H.L.); 2022205044@cqust.edu.cn (L.Z.); 2024205062@cqust.edu.cn (Y.L.); 2024205059@cqust.edu.cn (C.S.); 2025090120@cqust.edu.cn (S.Z.); 2 Chongqing Key Laboratory of Digitalization in Pharmaceutical Processes, Chongiqng University of Science and Technology, Chongqing 401331, China; 2003036@cqust.edu.cn (F.C.); chennan0205@cqust.edu.cn (N.C.)

**Keywords:** concave cubic gold, 6-deoxy-6-mercapto-β-cyclodextrin, paclitaxel, carboxymethyl chitosan

## Abstract

Paclitaxel is a first-line anticancer drug, but its low water solubility impedes bioavailability. The purpose of this study is to estalish a delivery strategy via carboxymethyl chitosan (CMCS)-encapsulated 6-deoxy-6-mercapto-β-cyclodextrins (dmβCDs)-modified concave cubic gold (CCGs) to achieve PTX release. CCGs were initially synthesized by the one-pot method and further modified by dmβCDs, the dmβCDs can effectively capture PTX molecules, followed by encapsulation with CMCS, and then prepare pH-responsive CMCS/dmβCDs/CCGs nanocarriers after lyophilization. Results indicated that desirable hexagonal CCGs with 50 ± 5 nm size can be obtained by adjusting H_2_O_2_ and HClO concentration. FT-IR, Raman and XRD spectra had confirmed dmβCDs successfully grafted to the surface of CCGs. Drug loading experiments demonstrated that the nanocarrier encapsulated PTX in amorphous powder or molecular form have a capacity of 55.05 µg/mL. Drug release experiments revealed PTX release from CMCS/dmβCDs/CCGs nanocarriers carrying a typical pH-responsive profile and indicating earlier release in an acidic environment than in a neutral or alkaline environment. The proposed method can be utilized to effectually achieve high-efficiency solubilization and targeted release inside tumor cells of PTX.

## 1. Introduction

Cancer with high morbidity and mortality is one of the major diseases that seriously threaten people’s health [1]. Chemotherapy is an effective way to treat cancer but many chemicals will bring severe side effects to patients [2,3]. On the other hand, the efficient delivery of drugs, especially poorly soluble drugs, is important for cancer treatment [4]. Therefore, it is essential to develop a novel drug carrier to promote their solubility. Paclitaxel (PTX), as a natural extract from *Taxus chinensis* [5], and its albumin conjugate [6] belong to the current first-line anticancer drug, which is one of the most frequently used agents for typical breast, ovarian, lung and colon cancer [7]. Unfortunately, the low solubility and bioavailability of PTX, as well as the side effects [8] via solvent dilution for intravenous infusion, limited its applicable potential [9]. Extensive research had mainly emphasized on the design of inorganic metal nanocarriers to achieve high-efficiency loading of PTX [10,11]. Gold nanomaterials are competent candidates due to their excellent water solubility and histocompatibility [12]. Previous studies primarily focused on gold nanoparticles [13], gold nanostars [14], and gold nanorods [15], which were modified by linker molecules on the material surface to enhance their interaction with PTX. but the solubilization of these methods is relative low-efficiency. Recently, there has been some progress of controllable synthesis of concave cubic gold with different morphology and surface structures [16], for example, the gold face of the gold cubic may be in (111), (100) or (110) growth directions showing specific atomic density, electronic structure and chemical reactivity [17]. They are particularly suitable for chemical linker molecular grafting [18]. But how to realize high-efficiency PTX-loading is still a very intractable problem.

Effective release of PTX from gold nanocarriers into cancer cells or target tissue is also another troublesome matter. It is technically necessary that the drug in a nanocarrier needs to be easily released without any loss of activity [13]. Compared with the ways of chemical coupling that will weaken its activity, inclusion by cyclodextrins (CDs) or a complex with phospholipid molecules [19] or polymer microsphere-based release [20] are more favorable to maintain its structure and activity. Additionally, the internal [21] or extracellular environment of cancer cells is more generally inclined towards acidic because of glycolytic cancer cell metabolism, hypoxia, and deficient blood perfusion [22]. Thus, priority consideration should be given for target release not in circulation by intravenous administration but after cell absorption in an acidic environment. Therefore pH-responsive materials are better candidates [23]. Carboxymethyl chitosan (CMCS) is a desirable pH-responsive material, which had once been reported in our previous work [24]. Hydroxymethyl and carboxyl methyl in the CMCS polymer make them easily form hydrogen bond networks that are prone to form gels or are easily collapsed by a weak acid environment [25]. This is very favorable for PTX release.

**Scheme 1:**
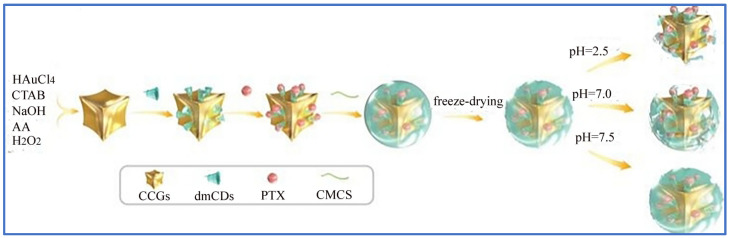
Illustration of carboxymethyl chitosan (CMCS)-encapsulated nanocarrier of 6-deoxy-6-mercapto-β-cyclodextrin-cyclodextrin (dmβCDs) grafted concave cubic gold (CCGs) for PTX delivery release under different pH conditions (pH = 2.5, 7.0 and 7.5). Where CCGs is concave cubic gold, dmβCDs is 6-deoxy-6-mercapto-β-cyclodextrin, PTX is paclitaxel, CMCS is carboxymethyl chitosan, AA is Ascorbic acid and CTAB is hexadecyl trimethyl ammonium bromide.

In contrast to hitchhiking on nanomaterials surface such as gold nanoparticles or nanorods that could destroy the PTX virgin structure [26] or only achieve low-efficiency loading (usually below than 20.0 µg/mL [27]), depth-tunable gold nanogroove cubic [28] can graft cyclodextrin to achieve high-efficiency loading of PTX. This study aims to develop a facile drug nanocarrier for PTX delivery. By regulating the reactant concentration during chemical synthesis to actualize accurate control of the groove’s curvature, CCGs with favorable indentations can then be utilized to load PTX via 6-Deoxy-6-mercapto-β-cyclodextrin (dmβCD) grafted in advance. pH-responsive CMCS was subsequently used to encapsulate the clinched PTX, resulting in the establishment of a comprehensive CMCS/dmβCDs/CCGs nanocarrier. The above-mentioned pH-responsive nanocarrier will easily be broken by the acid environment; thus, PTX will quickly release to the diseased sites of cells or tissues. Detailed design routine can be referred to Scheme 1.

## 2. Experimental

### 2.1. Reagents and Apparatus

Tetrachlororiform acid (HAuCl_4_), hexadecyl trimethyl ammonium bromide (CTAB),6-deoxy-6-mercapto-β-cyclodextrins (dmβCDs), hypochloric acid (HClO), 3-(4,5-dimethyl thiazole-2-group)-2,5-diphenyl-2h-tetrazolium-3-bromide (MTT), dimethyl Sulphoxide (DMSO), bovine serum albumin (BSA), penicillin, streptomycintrypsin, DMEM medium, and a dialysis bag (molecular cut off is 5000) were collectively purchased from Aladdin reagents in China (Aladdin, Shanghai, China) and used without further purification. Ascorbic acid (AA), hydrogen peroxide (H_2_O_2_), absolute ethyl alcohol and sodium hydroxide (NaOH) were all bought from Chuandong chemical Inc. (Chongqing, China). 4T-1 cells were offered by the laboratory animal center of Army Medical University in China [29]. UV-visible spectrophotometer (UV-2600, Shimadzu, Kyoto, Japan), Fourier transform-infrared spectroscopy (FT-IR, Tensor-27, Bruker, Billerica, MA, USA) instrument, X-ray diffraction (XRD, 7000S/L, Shimadzu, Billerica, MA, USA), Raman spectroscopy (LABHRAN HR Evolution, HORIBA, Kyoto, Japan), freeze dryer (FD-1D-50, Shanghai Bilon Instrument Co., Ltd., Shanghai, China), Laser granultometer (Mastersizer 3000E, Malvern Panalytical, Malvern, UK), Scanning Electron Microscope (SEM, JSM-7800, JEOL Ltd., Tokyo, Japan) and High-Resolution Transmission Microscope (HRTEM, JEM-2100F, JEOL Ltd., Tokyo, Japan) were equipped in CQUST. Deionized water (DO) was supplied in our Lab.

### 2.2. CCGs Synthesis by One-Pot Method

One-pot method was adopted to synthesize CCGs, according to the literature [18] but with slight modifications. Briefly, 206 μL of 24.28 mM HAuCl_4_ was added into 10 mL of 0.1 M CTAB solution and gently shaken. Subsequently, the above mixture was accurately adjusted to pH 11.46 by 1 M NaOH solution. Next, 11 μL of 30% H_2_O_2_ was added to the as-prepared mixture accompanied by vigorously agitation till to colorless. The final reaction solution was placed in a 30 °C water-bath for 15 min incubation after addition of 300 μL AA (0.1 M) and 30 s of rigorous stirring.

### 2.3. Termination of Synthesis Reaction

20.0 μL of 1 M HClO solution was well-distributed into the as-mentioned CCGs solution (absorbance of UV value is 0.3) to consume excess AA and H_2_O_2_ to terminate the chemical reaction, maintaining CCGs’ moment configuration. In total, 10.0 mL of the as-prepared CCGs solution were 12,000 rpm centrifugated under room temperature. Recovered CCGs precipitates were uniformly dispersed by DO and preserved in 4 °C refrigerator for later characterizations.

### 2.4. Dmβcds Graft to CCGs

A certain volume of dmβCDs solution was mingled with 3.0 mL CCGs solution under 10 °C by 180 rpm agitation to react 60 h, for exploring the influences of dosage, reaction times, temperatures and rotation rates to prepare dmβCDs/CCGs nanocarrier.

### 2.5. Plotting Standard Curve of Ptx

In total, 0.1 mg/mL of Ptx standard solution (Sd Solution) was prepared in advance by 0.585 Mg Ptx dissolved in 95% ethanol solution with a final constant volume of 50 mL. 3.0 mL serial diluted Sd solutions were in proper order and scanned by Uv Spectrometer to confirm the absorbance values. Then, plot when the absorbance values were defined as ordinate and then concentrations of Ptx/ethanol as horizontal and fit for the corresponding function relationship.

### 2.6. Dmβcds/ccgs Nanocarrier Loading Ptx

First of all, 1.32 mL 10 mM PTX standard solution was dropwise added into dmβCDs/CCGs solution (dmβCDs:PTX = 1:5, m/m) for 24 h reaction at room temperature. Then, recover the purple precipitates by 8000 rpm and 5 min centrifugation. Loading percentage of PTX was thereupon calculated on the basis of solubility curve after deduction of the supernatant reckoned from UV measurement.

### 2.7. Ptx Release from Cmcs Wrapped Dmβcds/ccgs Nanocarrier

We weighed a specific mass of CMCS and dissolved it in the pre-fabricated CMCS/dmβCDs/CCGs mixture to prepare solutions ensuring CMCS concentrations of 50 mM, 100 mM, 150 mM, 200 mM, and 250 mM, respectively. After homogenization, the mixture was lyophilized at –30 °C for 48 h to obtain CMCS/PTX/dmCDs/CCGs solid dispersoids. The solid dispersoids were separately dialyzed in activated dialysis bag at pH 2.5, 7.0 and 7.35, which were orderly placed in 80 mL PBS buffer under 37 °C with 120 rpm agitation. Interval sampling at 3 mL was conducted and equal volume of PBS was quickly supplemented to the above solution. The absorbance of the acquired samples was measured by UV at 233 nm. Therefore, cumulative release curves of PTX were obtained from the plotting when T is designated as horizontal ordinate and cumulative release rate as longitudinal coordinates. The formula is as follows:

(1) $$A\;=\;38.177C\;+\;0.037\;(R^{2}\;=\;0.999)$$

(2) $$E_{r}=\frac{V_{e}\sum_{i}^{n-1}C_{i}+V_{0}\;\times \;C_{n}}{m_{\mathrm{drug}}}$$

*E_r_*: Cumulative release volume,

*V_e_*: Buffer displacement volume,

*V_0_*: Total volume of release medium,

*C_i_*: Concentration of the release fluid at the i-th displacement sampling,

*m_drug_*: Total mass of the drug loaded in the vehicle,

*n*: Number of substitutions of the buffer

### 2.8. Cell Viability Assay

Mammary cancer cells 4T-1 from a refrigerator set to −150 °C were quickly revived in a 37 °C water-bath for 1 min. Revived cells were immediately placed in DMEM medium and thereafter transferred into centrifuge tube for 5 min centrifugation. Then, discard the supernatant, add a small amount of medium into the tube, then lay it in an incubator to perform a morphology check. Culturing conditions are 37 °C, 5% carbon dioxide, 95% humidity, addition of 10% FBS, 100 U/mL penicillin and 100 U/mL streptomycin. The cells cultured all the time until they reached 80% anchorage rate after 0.25% trypsin digestion. The cultured cells were adjusted to 5 × 10^3^/mL. Six groups of 200 microliters per well were simultaneously inoculated in four 96-well microplates with five wells in each group, keeping identical culturing condition. Five groups of each 96-well plate were randomly selected to add the final concentration of each experimental drug, while blank controls include DMSO and culture medium. Approximation 4 h at the end of the culture, 20.0 μL MTT (5.0 mg/mL) was added to each well. After 150 μL 10% DMSO was added, cell suspension in microplates were uniformly mixed and measured by UV (OD_492_). The inhibition of cell proliferation was calculated according to the following formula. Inhibition rate% = [1 − (OD_experimental well_/OD_control well_)] × 100%.

### 2.9. Molecular Simulation

Both PTX and dmβCDs were processed using Autodock tools 1.5.6 software and nonpolar hydrogen atoms were merged and saved in pdbqt format. Combination of PTX and dmβCDs was performed by blind docking with Autodock Vina software to obtain the optimal binding conformation. The size of the grid calculation is 16 Å × 12 Å × 16 Å; the grid space is 1 Å. Exhaustiveness is set as 200 times, producing 20 binding conformations. Other parameters are set to default values. Finally, the optimal docking conformation of the PTX and dmβCDs complex was selected for binding energy and root mean square deviation (RMSD).

### 2.10. Characterization

(1) Ultraviolet visible (UV-vis) measurement. A certain amount samples were dissolved in suitable solvent and DW as a blank control. Then, using the double beam UV photometer, the samples were scanned within wavelength of 200–100 nm to get UV spectrogram. (2) Fourier transform-infrared spectroscopy (FT-IR) characterization. Samples were mixed with KBr (m/m = 1:10) and delivered for tablet compressing. Nicolet 10 (Thermo Nicolet, Waltham, MA, USA) was used for measurement. The scanning wavelength is from 4000 to 400 cm^−1^. (3) X-ray diffraction (XRD) characterization. Crystal structures of samples were determined by an X-ray diffraction analyzer (Shimadzu, Japan XRD-70000). The scanning range is 5~60 and the speed is 5/min. (4) Scanning Electron Microscope (SEM) and transmission electron microscope (TEM) observation. mSEM and TEM images of the prepared materials were taken on a JEOL JEM2010 microscope operating at 200 kV. (5) Raman spectroscopy data were collected with a DXR Raman microscope (Thermo Fisher Scientific, Waltham, MA, USA) system by exciting with the 532-nmlineo fan Arion laser. Exciting power at the sample was 7 mW, with a typical exposure time of 12 s and at least 10 repetitions. The scattered light collected was analyzed on a triplet spectrograph at intervals of 1.0 cm^−1^ with an integration time of 1.5 s. The system is equipped with a 900-lines-per-millimeter holographic grating and a spot size of 0.7 mm slit. A cooled charge-coupled device detection system was used to acquire Raman data, captured with a single exposure. All Raman spectroscopy measurements were performed at 21 °C shortly after sample preparation. (6) Potentiometry determination. Rinse the Marvern cuvette with ethanol twice, then transfer 1 mL of the sample into the Marvern cuvette (Thermo Fisher Scientific, Waltham, MA, USA). Each sample was measured three times.

## 3. Results and Discussions

### 3.1. Synthesis of CCGs

**Figure 1:**
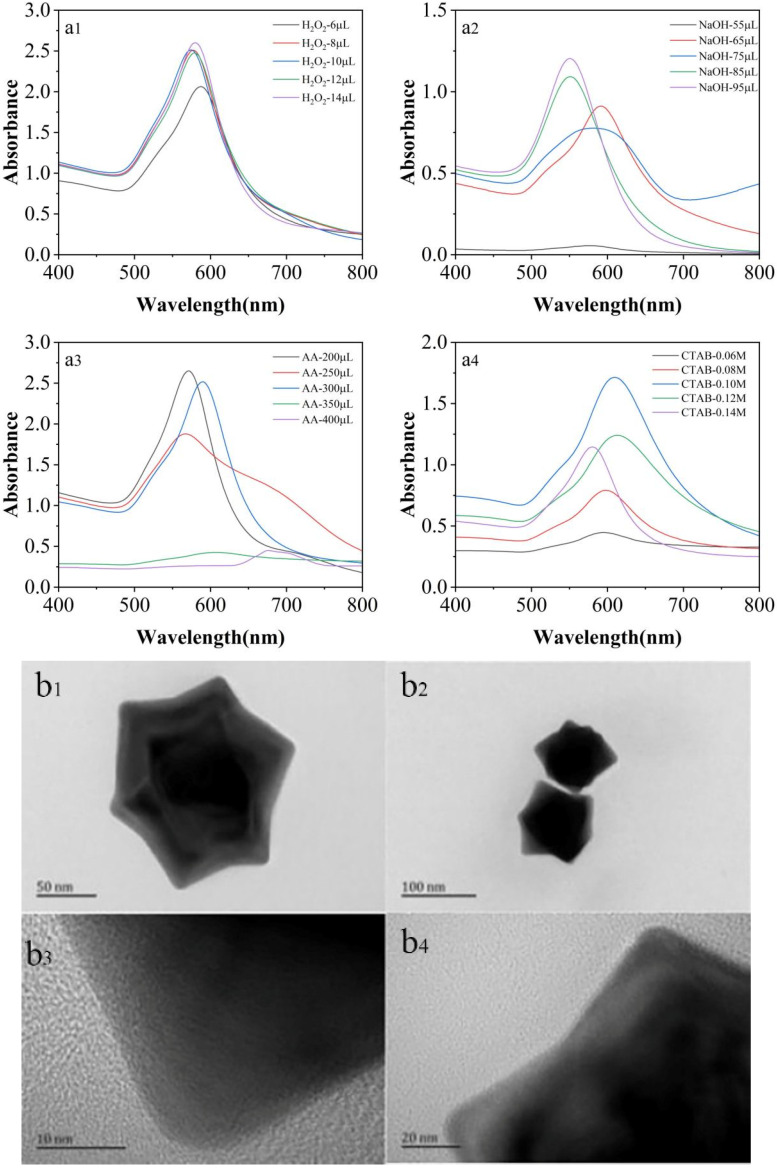
UV spectra of reactant concentration after optimized dosage of H_2_O_2_ (**a1**), NaOH (**a2**), AA (**a3**) and CTAB (**a4**) together with the corresponding optimal TEM images (**b1**–**b4**) of CCGs.

Recently, gold nanomaterials have been frequently used for development of drug carriers due to their excellent histocompatibility [12]. Gold nanomaterials, especially for CCGs, are gradually emphasized as their controllable preparation and exposed surfaces [30] that can be easily used for application of drug carriers. The CCGs were synthesized by one-pot method carried dark blue color and represented maximum UV absorption peak at 596 nm together with 530 nm shoulder peak (longitudinal and transverse peaks), which exist 54 nm differences with reported data [31]. The results from optimized reactant concentration shows that high H_2_O_2_ content will strengthen reducing force to Au^3+^, leading to smaller size of CCGs (Figure 1(a1)). The reaction system was stabilized by alkaline environment that 65 μL dosage of NaOH can get optimum results (Figure 1(a2)). Meanwhile, AA can guide gold reduction and longitudinal growth. Higher AA concentration, the sharper point of CCG’s edge, even grow into irregular shape particles when they gradually gathered to a certain extent (Figure 1(a3)). As a surfactant, CTAB plays both a stable reagent and a structure-oriented role in the process of CCGs synthesis; its absence will induce reactant aggregates or instable system at low concentration (Figure 1(a4)). Further studies demonstrated that water-bath time of less than 15 min for CCGs will develop insufficient growth, but more than 20 min will result in irregular spherical or elliptical shapes. Based on the optimized reaction condition, 6 μL H_2_O_2_, 65 μL NaOH, 300 μL AA, 0.1 M CTAB and water-bath of 15 min can achieve desirable CCGs (Figure 1(b1–b4)) geometrical configuration.

### 3.2. Reaction Termination and Recovery of CCGs

**Figure 2:**
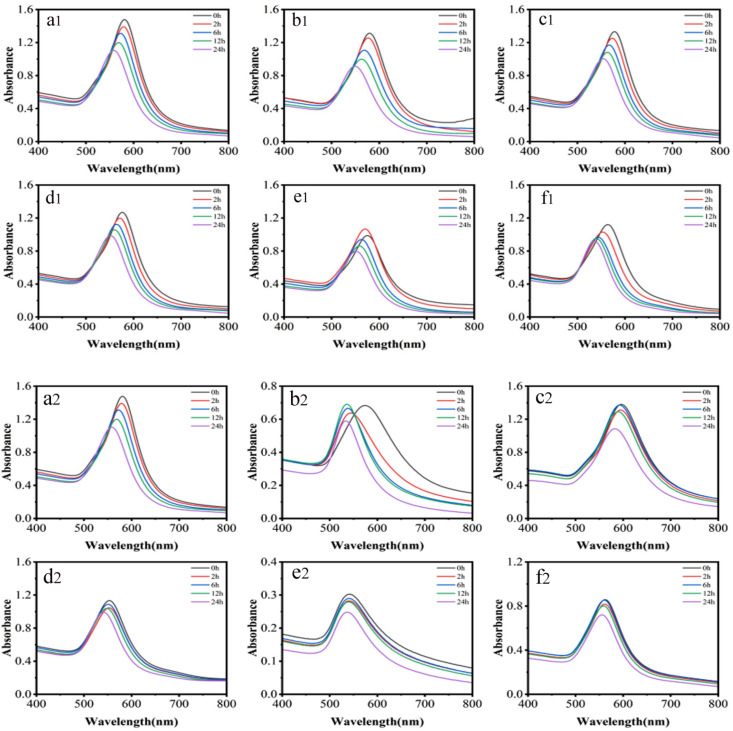

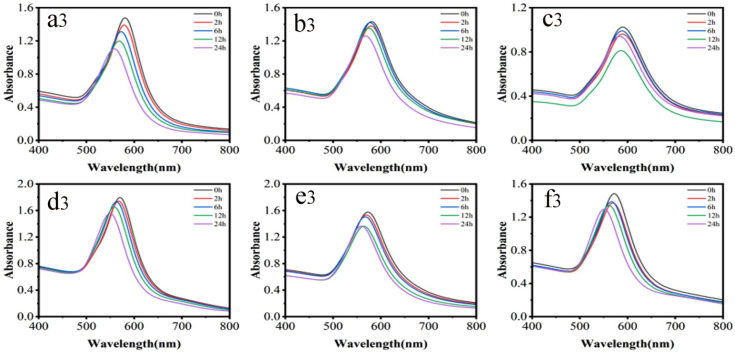
UV spectra of CCGs by addition of 0 μL (**a1**), 100 μL (**b1**), 200 μL (**c1**), 300 μL (**d1**), 400 μL (**e1**), 500 μL (**f1**) HClO solution, reaction temperature 4 °C (**a2**), 10 °C (**b2**), 14 °C (**c2**), 18 °C (**d2**), 22 °C (**e2**), 26 °C (**f2**) and placement time 0 h (**a3**), 2 h (**b3**), 4 h (**c3**), 6 h (**d3**), 12 h (**e3**) and 24 h (**f3**) after 12,000 rpm centrifugation.

Actually, CCGs synthesis in whole is a gradual process, from the initial gold nanoparticle seed to the formation of the gold cubic, to the excess reactant in the reaction system, that will affect its curvature shape formation, which is not conducive to follow-up drug loading [30]; thus, acquisition of the ideal curvature of CCGs requires accurate control of the reactant dosage and precise regulation of the reaction condition. Furthermore, suspended solutions must be taken to stop the reaction once the gold nanogroove is formed. In our experiments, we found that the CCGs reaction system was greatly affected by H_2_O_2_ and AA concentration, and if the redox reaction was not terminated, UV spectra displays a red shift, indicating that large gold nanoparticles were generated. Therefore, we systematically investigated that HClO was designated as a discontinuous agent to stop the reaction for the acquisition of excellent curvature of CCGs. Compared with blank control, addition of 100 μL, 200 μL, 300 μL, 400 μL and 500 μL HClO can effectively terminate chemical reaction by consuming the unreacted H_2_O_2_ and AA, as certified by the UV results of Figure 2. It must be noted here that excessive HClO will conversely etch the synthesized CCGs into nanoparticles or other irregular shapes. Moreover, all operations must be operated at low-temperature conditions to prevent the bad effects of higher temperatures. It finally proved that satisfactory CCGs products could be obtained by centrifugation at 14 °C with 50 μL HClO dose.

### 3.3. Regulation of Ccg’s Curvature

**Figure 3:**
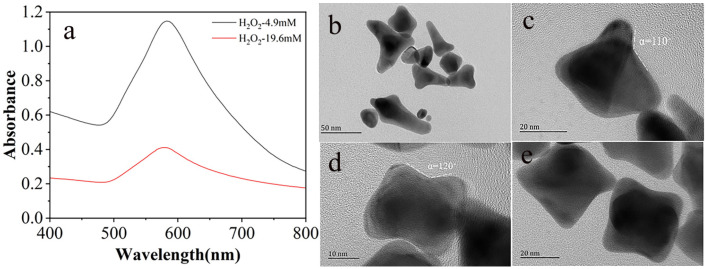
UV spectra (**a**) and TEM images of CCGs for H_2_O_2_ concentration of 4.9 mM (**b**,**c**) and 19.6 mM (**d**,**e**). The angle indicates the width and depth of the groove of CCGs.

**Figure 4:**
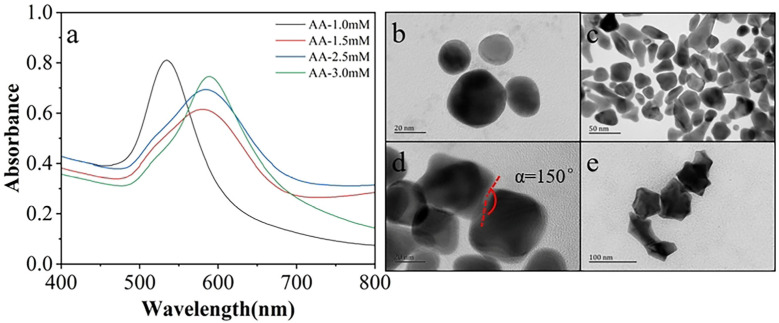
UV spectra (**a**) and TEM images of CCGs under AA concentrations of (**b**) 1.0 mM, (**c**) 1.5 mM, (**d**) 2.5 mM, and (**e**) 3.0 mM.

Among different shapes, concave nanomaterials have emerged as a novel class of unconventional materials with superior properties [28]. Clearly, a deeper curvature of CCGs needs to be adjusted to facilitate drug loading. Regarding the synthesis of CCGs, it is generally believed that H_2_O_2_ reduces Au^3+^ to Au^+^ in CTAB solution and the addition of AA can facilitate the nucleation of gold crystal and symmetry breaking formation of CCGs [31] at specific sites by color changes from yellow to colorless, which will be reinforced by alkaline conditions (pH > 7). Consequently, increasing H_2_O_2_ concentration can reduce the degree of concave curvature (Figure 3) while increasing the AA can increase it (Figure 4). When the concentration of AA is 1.0 mM, 1.5 mM, 2.5 mM and 3.0 mM, the solution has maximum UV absorption peaks at 534 nm, 579 nm, 589 nm and 579 nm, and a transverse peak around 520 nm by 1.5 mM and 2.5 mM is clear evident. But 20–40 nm nanoparticles with circular edges were occasionally obtained according to the UV curve indication (Figure 4c). More irregular shapes, such as tripods, four tripods, spheres, cubes, and branched nanoparticles, were also unexceptionally discovered when AA concentration was higher than 1.5 mM (Figure 4d). CCGs can achieve a 30–40 nm regular morphology with 2.5 mM concentration of AA and even gain 50–80 nm hexagonal configuration with 3.0 mM concentration of AA (Figure 4e). From the measurements, approximately 150 degree of curvature can be well-defined.

### 3.4. Dmβcds Graft CCGs

**Figure 5:**
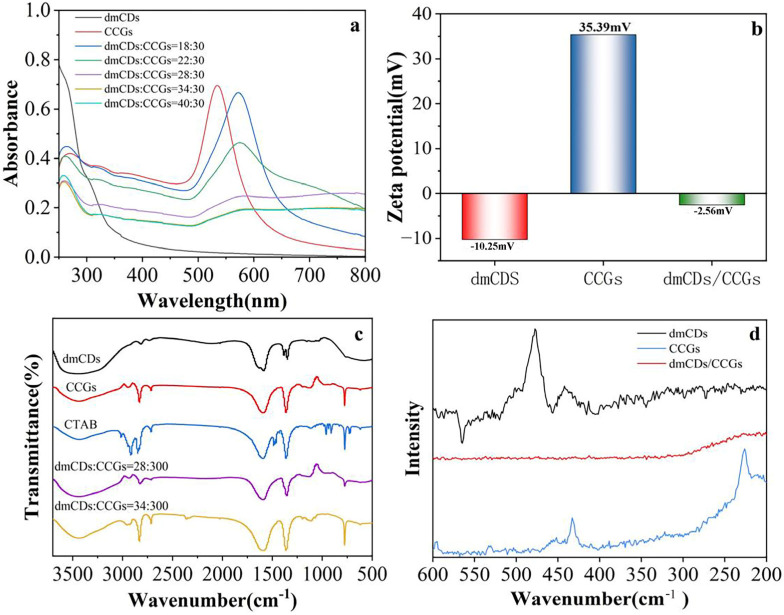
UV spectra (**a**) of dmCDs, CCGs and different mass ratio, potential (**b**) with dmβCDs, CCGs and dmβCDs/CCGs, FT-IR (**c**) of dmβCDs, CCGs, CTAB and dmβCDs/CCGs (mass ratio 28:300 and 34:300) and Raman spectra (**d**) of dmβCDs graft to CCGs.

In this study, we used dmβCDs to graft CCGs as a kind of rivet for the subsequent fixation of PTX. According to the literature [32], sulfur forms a self-assembled monolayer membrane on the surface of gold atoms, luring gold atoms to form a chain or a monolayer-protective cluster. Gold atoms in the center of the gold nanocluster are deemed as the core, and sulfur-containing substances from the monolayer protective film on the core surface finally evolve from ring (ring Au) or chain (Au chain) to a strong Au-S bond [33]. The UV spectra (Figure 5a) together with its magnification image (Figure 5c) revealed that application of 220 μL 10 mM dmβCDs under 0 rpm, 40 rpm, 80 rpm and 160 rpm agitation reaction can get maximum UV absorption peaks at 563 nm, 573 nm, 573 nm, 573 nm, 574 nm and 573 nm, respectively, in addition to an additional 10 nm red shift. The prepared dmCDs/CCGs nanocarrier takes negative charge −2.56 mV in comparison to −10.25 mV of dmβCDs and −35.35 mV of CCGs alone (Figure 5b). Except observable evidence of FT-IR spectrum that retained sulfhydryl peak of dmβCDs (Figure 5c), which can testify to the formation of coupling between dmβCDs and CCGs, there was a palpable Raman shift at 236 nm that can be attributed to the formation of Au-S bond [18]. Considering that the subsequent package cooperation among dmβCDs to PTX may involve steric hindrance effects, the dosage ratio between them was finally determined to be 1:12 after theoretical estimation and experimental optimization, expecting acquisition of free PTX molecules protrusion on the CCGs surface. Therefore, the optimal condition of grafting reaction is identified as 220 μL dmβCDs, 8 °C and 120 rmp agitation with 12 h reaction.

### 3.5. Dmcds Clinch Ptx

**Figure 6:**
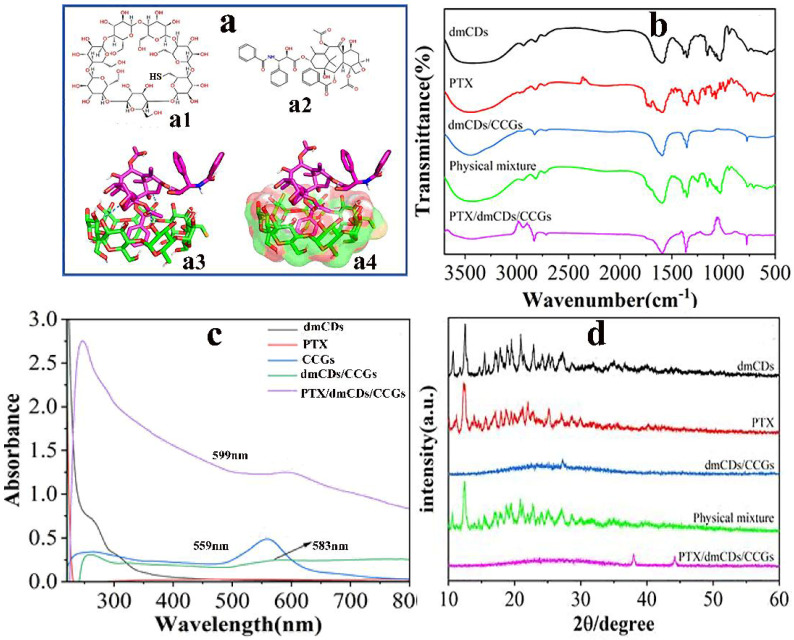
Theoretical simulation of inclusion interactions (**a**) between dmCDs (**a1**), PTX (**a2**), molecular docking conformation diagram (**a3**) and molecular docking surface diagram (**a4**) that was demonstrated by FT-IR (**b**), UV (**c**) and XRD (**d**) characterization; detailed descriptions are seen from the text.

Noncovalent interactions involved with H-bonding, van der Waals forces, electrostatic interactions, and hydrophobic interactions are usually used to drive molecule interaction [34]. Such interaction, especially inclusion action by CDs, can lead to the specific immobilization of insoluble drugs [35]. PTX cannot fully enter into the hydrophobic cavity of dmβCDs [36]. It is supposed that dmβCDs can only clamp a specific hydrophobic group of PTX and rivet the PTX molecules through hydrophobic interaction. When PTX approaches the cavity of sulfhydryl-β-cyclodextrin, the electrons of CCGs are further dispersed, causing red shift on the UV spectrum. According to the density functional theory, only parts of PTX (3 benzene rings) are wrapped into the hydrophobic cavity of CDs [36] (Figure 6a). Interactions between the remaining hydrophobic groups caused dmβCDs/CCGs aggregation, meanwhile leading to purple flocculent aggregates in the solution. PTX was delivered into dmβCDs/CCGs solution for 24 h reaction to get PTX/ dmβCDs/CCGs inclusion. The UV maximum absorption peak of the latter is shifted to 598 nm (Figure 6b) compared with 570 nm of the former. The FT-IR spectra (Figure 6b) indicate that PTX/dmβCDs/CCGs display typical hydroxy characteristic peaks of 3439 cm^−1^ with blue shift from 3491 cm^−1^ to 3444 cm^−1^, compared with that of dmβCDs, resulting from formation of hydrogen bonds between dmβCDs and PTX, which shows weakened characteristic peaks at 1715 cm^−1^ and 1739 cm^−1^ of acetyl together with obvious difference of fingerprint region at 600–1300 cm^−1^ compared with that of the physical mixture. The UV spectrum indicates that the maximum ultraviolet absorption peak of dmβCDs/CCGs is at 583 nm. After loading PTX, the absorption peak shifts red-shifted to 599 nm with increased absorbance, confirming successful drug inclusion (Figure 6c). Other evidences of successful inclusion interaction are that XRD of PTX/dmCDs/CCGs only retained relatively weak 37.98° and 44.22° peaks (Figure 6d) but dmβCDs possess sharp-pointed 10.72° and 12.56° peaks. In addition, PTX holds an intense 12.34° peak besides simple peaks overlying of physical mixture; this inferred that dmβCDs/CCGs is amorphous.

### 3.6. Cmcs Encapsulate Ptx/dmβcds/ccgs

After inclusion of PTX by dmβCDs/CCGs, CMCS was applied to wrap the PTX/dmβCDs/CCGs for preparation of a CMCS/PTX/dmβCDs/CCGs pH-responsive drug carrier. The encapsulation rate is thereafter calculated. Unwrapped PTX (where absorbance of the supernatant is 0.317 at 233 nm) was first calculated. Encapsulation rate (ER) could be inferred from Equation (3) as following:

(3) $$ER=\frac{m_{\mathrm{PTX}\;\mathrm{input}}-m_{\mathrm{PTX}\;\mathrm{in}\;\mathrm{supernatant}}}{m_{\mathrm{PTXinput}\;}}$$

**Figure 7:**
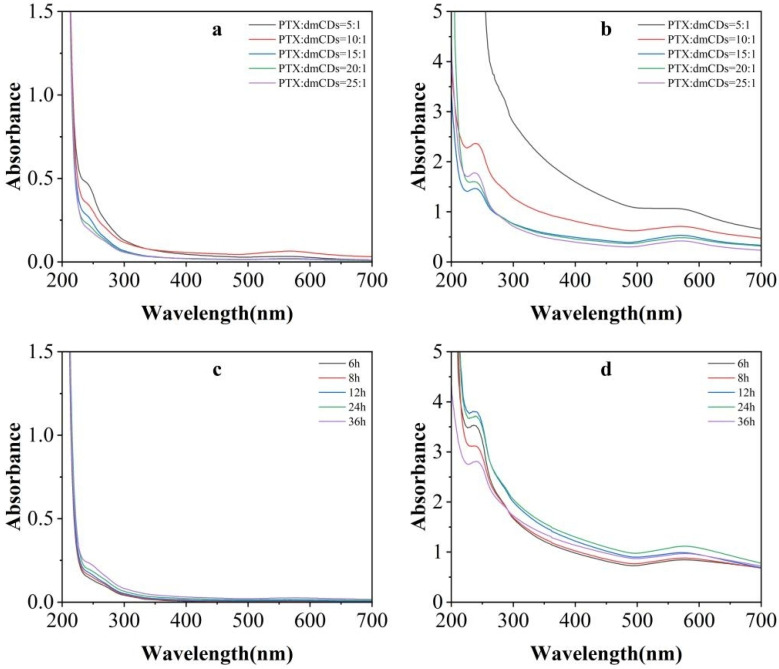
UV of centrifuged supernatant (**a**), resuspension of centrifuged precipitate (**b**) for 1:5, 1:10, 1:15, 1:20, 1:25 admixture and of centrifuged supernatant (**c**), and resuspension of centrifuged precipitate (**d**) for 1:10 mixture after reaction of 6 h, 8 h, 12 h, 24 h and 36 h.

The ER is 92.36% that is remarkably higher than the reported polymeric nanocapsule of 83.8 ± 4.7% [20]. The highest content of PTX in the proposed nanocarrier can reach up to 55.05 µg/mL, which is much higher than that of PTX alone (only 0.4 µg/mL) or the prodrug [37]. More incubation time shows no significant change for PTX content in the supernatant but prominent increase of PTX in sediment resuspension. There was no red or blue shift of characteristic absorption peaks (Figure 7a) for both PTX (233 nm) and dmβCDs (570 nm), which confirmed the formation of stable inclusion. ER at that time were 92.36%, 91.75%, 91.06%, 89.98% and 88.82% received. This further demonstrates that stirring is detrimental to the formation of inclusion bodies. It is hypothesized that the inclusion bodies form a multilayer structure on the surface of CCGs rather than a monolayer due to the hydrophobic nature of PTX (Figure 7b). Analogous experiments further examined the different molar ratios (mole ratio of 1:5, 1:10, 1:15, 1:20 and 1:25) of PTX/dmβCDs. The absence of dmCDs UV absorption peaks and the sharp reduction of PTX UV absorption at 233 nm both demonstrated that PTX in the supernatant was present as free molecules not bound ones (Figure 7c) This is by and large in accordance with the expectations monitored in sediment suspension (Figure 7d). The characteristic absorption band of both of them are red shifted. High concentrations of PTX tended to be more turbid in the solution. ER of 90.32%, 85.16%, 85.52%, 81.96% and 79.47% at corresponding mole ratio were deduced. It is noteworthy that the encapsulation rate is highest at PTX:dmβCDs = 1:5; as such, the UV peak of PTX masked the UV peak of dmβCDs, indicating that some PTX were not incorporated into dmβCDs, rather than hydrophobically clustered on the surface of dmβCDs. The UV peak of dmβCDs can be clearly observed when PTX:dmβCDs = 1:10, indicating that PTX as an inclusion complex at this concentration resulting from both the curvature of the CCGs and steric configuration gives rise to multiple dmβCDs molecules for PTX cooperative wrapping.

### 3.7. pH-Responsive Release of Ptx from Cmcs/dmβcd/ccgs Nanocarrier

**Figure 8:**
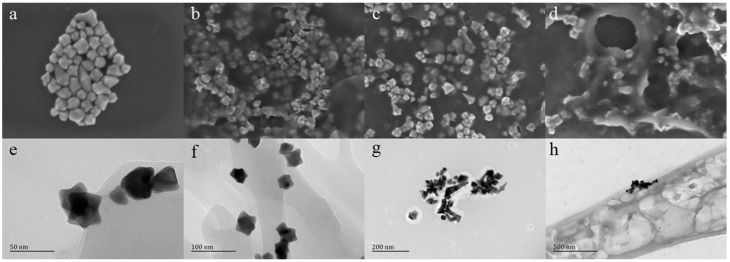
SEM of native CCGs (**a**), dmβCDs grafted CCGs (**b**), PTX/dmβCDs/CCGs inclusion (**c**), CMCS/PTX/dmβCDs/CCGs nanocarriers (**d**) and TEM of PTX/dmβCDs/CCGs (**e**), CMCS/PTX/dmCDs/CCGs at pH 2.5 (**f**) and 7.0 (**g**) together with that at pH 7.35 (**h**) for 24 drug releasing, respectively.

Nanotherapeutics demonstrated that poor accumulation of the drug in the tumor microenvironment may bring about inferior discharge and release into the tumor cells [26]. Therefore, effective release of nanocarrier-loaded drugs is the primary prerequisite for drug entry into cells. Considering the fact that the intracellular environment is acidic, a preferable strategy to use CMCS material as a wrapper is of particular interest. In the designed experiment, as a typical ion-responsive material [25], CMCS has excellent gel formation properties; hence, it is ideally used as an ion-responsive sealer. The drug release behavior of the pH-responsive CMCS/dmβCDs/CCGs nanocarrier was evaluated by in vitro assays. Figure 8 displays SEM images of native CCGs (a), CCGs grafted dmβCDs (b), PTX-fixed dmβCDs/CCGs inclusion bodies (c), and CMCS-coated PTX/dmβCDs/CCGs complexes (d). Absorption values of a series dilution of PTX (0~0.025 mg/mL)/ethanol solution were measured by UV-vis and PTX linear concentration/absorbance was plotted by standard curve: A = 38.177C + 0.037 (R_2_ = 0.999, C is concentration of PTX). Release behavior of CMCS/dmβCDs/PTX/CCGs was investigated in PBS buffer at pH 2.5, 7.0, and 7.35, respectively.

The CMCS has a very strong water absorption ability [38]. At pH = 2.5, it was presumably inferred that CMCS/PTX/dmβCDs/CCGs does not absorb enough water leading to only achievement of drug release by breaking the CMCS gels on account of the buffer, which has a large inorganic salt ion concentration. The main influencing factors of this kind of release mechanism are attributed to the pH and the concentration of CMCS. When CMCS concentration is no less than 100 mM, the pH effect dominates the gel disintegration. The lower the pH value, the faster the swelling and rupture of the water-absorbent gel, resulting in greater cumulative drug relea as shown in Figure 8e (freshly prepared PTX/dmβCDs/CCGs) and Figure 8f (PTX release from CMCS/PTX/dmCDs/CCGs). When CMCS concentration is greater than 100 mM, the concentration influence of CMCS is dominant. Higher CMCS results in more brittle gels. The collapse process of CMCS gel amplified larger accessible surface area of PTX that mediated greater cumulative release rate while PTX demonstrated a poor release. It must be noted here that pH-responsive CMCS rupture highly favors PTX exposure to intracellular environment and strengthen its pharmacotherapeutic effects at specific sites. This is no doubt very propitious to cancer cell-related disease therapy that is usually considered to be an acidic environment.

**Figure 9:**
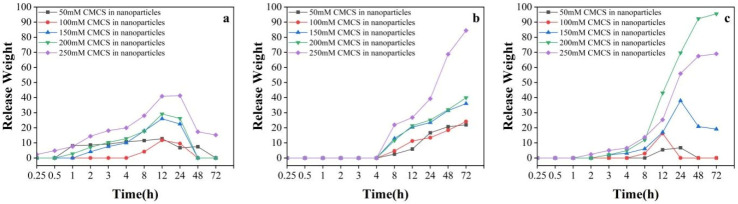
UV spectra of 50 mM, 100 mM, 150 mM, 200 mM and 250 mM CMCS in /PTX/dmβCDs/CCGs nanocarrier at pH = 2.5 (**a**), pH = 7.0 (**b**) and pH = 7.35 (**c**) in PBS buffer for 15 min, 30 min, 1 h, 2 h, 3 h, 4 h, 8 h, 12 h, 24 h, 48 h and 72 h release together with the cumulative release rates.

Samples prepared by freeze-drying with solvents of different properties exhibit distinct water absorption processes. At pH = 7.0, in spite of PTX release abiding by the aforementioned style, there emerged a different release mode after the 24 h record. No PTX was detected within two hours (Figure 8f). The above analysis supports the hypothesis that low-concentration CMCS of CMCS/PTX/dmCDs/CCGs complexes will rapidly swell [25] due to excessive water absorption due to fewer inorganic salt ions, subsequently transforming into sols and forming a dilution-release model. The purpose of drug release is achieved through gently stirring and shaking, but the volume of CMCS swelling is limited by the dialysis bag volume, and the dilution-release mode is mainly affected by CMCS concentration under the condition of weak dissociation. This also explains why there is a more explosive release and larger cumulative release rate after 24 h than that at pH = 2.5. Conversely, there was almost no PTX release for all diluted CMCS/PTX/dmβCDs/CCGs within 4 h when pH is at 7.35 (Figure 8h). After that, the CMCS/PTX/dmCDs/CCGs nanocarrier began to swell under stirring conditions and the cumulative release rate of PTX with a moderate rate ultimately reached 64.46%. The effect of thicker layers caused by CMCS dosage on PTX release under different pH conditions is shown in Figure 9. It shows that this system only exists dilution-release mode without gel-fracture release as there is no UV monitored absorption peak and the nanocarrier is more stable in an alkaline environment than that in an acid or neutral environment. Apparently, such a low release rate under alkaline conditions is very favorable for normal cells because intracellular environment usually maintains a weak alkaline environment [39].

### 3.8. MTT Assay

**Figure 10:**
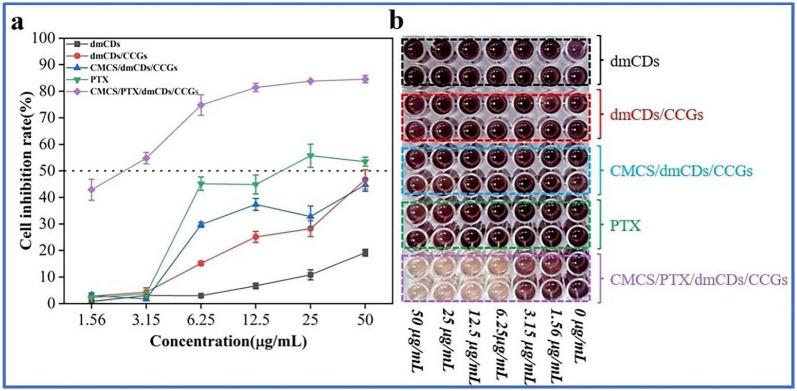
Cell inhibition rates (**a**) and color aspects (**b**) of breast cancer 4T1 cells administered via addition of dmCDs, dmCDs/CCGs, CMCS/dmCDs/CCGs, PTX and CMCS/PTX/dmCDs/CCGs agentia by MTT determination.

The MTT is a yellow water-soluble tetrazolium salt that can be reduced as purple methyl azo bridge (formazan) precipitate when it was added to metabolic active cells [40]. Figure 10 displays the results for the rate of cell growth inhibition (Figure 10a) and color aspects of cell precipitate in microplate (Figure 10b) of 4T1 cells with drug treatment by MTT assay. Comparatively speaking, dmCDs group showed a mere minimum inhibition rate compared to the PTX, dmCDs, dmCDs/CCGs, CMCS/dmCDs/CCGs and CMCS/PTX/dmCDs/CCGs groups, even holding 20% and no detectable IC_50_ using a 50.0 µg/mL dose. All experimental groups represent dose-dependent effect. IC_50_ of them were 50.00 µg/mL, 50.00 µg/mL, 25.00 µg/mL, and 3.15 µg/mL, respectively. Namely, the IC_50_ of proposed PTX-loaded nanocarriers were nearly 16-fold higher than that of PTX alone. The results proved that the developed nanocarrier has outstanding PTX delivery, release and anticancer effects for cancer cells.

## 4. Conclusions

Solubilization of insoluble drugs remains a persistent challenge. This study proposed a new strategy to construct a facile nanocarrier for PTX delivery. The strategy involves site-directed grafting dmCDs onto the one-step synthesized CCGs surface through Au-S bond linkage to anchor PTX via hydrophobic interactions, followed by coating with CMCS and free-drying treatment. The large cavity formed by the tunable curvature of CCGs enables high drug loading capacity of PTX compared to conventional surface-mounted methods, which can be up to 55.05 µg/mL. The prepared CMCS/dmβCDs/CCGs nanocarriers exhibit typical pH-responsive characteristics. Loaded PTX between CCGs and CMCS can be adequately released with a cumulative release rate of 92.26% when pH is lower than 7.0 or slightly released with a cumulative release rate of 64.46% when pH is higher than 7.0, relying on the encapsulated thickness of CMCS gel and solvent properties. The MTT assay shows that IC_50_ of proposed PTX-loaded nanocarriers were nearly 16-fold higher than that of PTX alone. In summary, the proposed strategy of establishment of the PTX nanocarrier can effectively achieve solubilization via adjusting the depth of CCGs and quantity of CMCS encapsulation, which can be utilized to efficiently clinch insoluble PTX without compromising its structure and activity for subsequent successful pH-responsive release.

## Data Availability

The original contributions presented in this study are included in the article. Further inquiries can be directed to the corresponding author.
